# Miao Sour Soup Modulates Hepatic Gluconeogenesis Through PI3K/Akt/Foxo1 Signaling in High‐Fat Diet–Induced Rats

**DOI:** 10.1002/fsn3.71900

**Published:** 2026-05-20

**Authors:** Xue Ao, Xiaoying Liu, Qin Yuan, Xuan Guo, Hangning Qian, Huihui Li, Huiqun Wang

**Affiliations:** ^1^ School of Public Health, the Key Laboratory of Environmental Pollution Monitoring and Disease Control, Ministry of Education Guizhou Medical University Guiyang China; ^2^ Guizhou Techonicial Innovation Center of Suantang Guiyang China

**Keywords:** chronic inflammation, hepatic gluconeogenesis, insulin resistance, Miao Sour Soup, PI3K/Akt/FoxO1 signaling pathway

## Abstract

Miao Sour Soup (MSS) is a traditional fermented dish containing bioactive constituents such as lycopene, capsaicin, and short‐chain fatty acids, which have been associated with anti‐obesity, anti‐inflammatory, and antioxidant effects. Excessively elevated hepatic gluconeogenesis is a critical element instigating energy metabolism dysregulation in the body. This research examined the extent to which MSS enhances energy metabolism abnormalities caused by a high‐fat diet via hepatic gluconeogenesis and confirmed this route. The findings indicated that MSS intervention markedly decreased hepatic lipid accumulation, pro‐inflammatory cytokine secretion, and blood lipid and glucose levels in rats subjected to a high‐fat diet. Non‐targeted metabolomics study revealed that MSS influences energy metabolism pathways, including glycolysis and gluconeogenesis. Subsequently, Western blot analysis was conducted to assess protein expression in the NF‐κB inflammatory pathway and the PI3K/Akt/Foxo1 signaling pathway. The findings indicated that MSS decreased NF‐κB expression in the livers of rats subjected to a high‐fat diet, thereby activating the PI3K/Akt/Foxo1 signaling pathway and decreasing the expression of PEPCK and G6PC, essential proteins in gluconeogenesis. This study's results demonstrate that Foxo1 is a crucial protein in the dysregulation of glucose metabolism caused by lipid metabolism diseases. MSS may stimulate the PI3K/Akt/Foxo1 signaling pathway by mitigating chronic inflammation caused by a high‐fat diet, therefore inhibiting excessive hepatic gluconeogenesis and ultimately improving insulin resistance.

## Graphical abstract

Miao Sour Soup may mitigate chronic inflammation caused by a high‐fat diet, consequently activating the PI3K/Akt/Foxo1 signaling pathway, downregulating PEPCK/G6PC expression, and finally suppressing the aberrant increase of hepatic gluconeogenesis while improving insulin resistance.

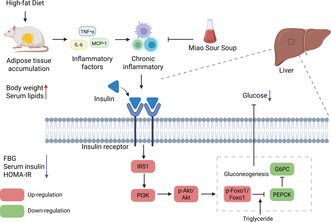

## 1 Introduction

Hepatic gluconeogenesis, a crucial physiological mechanism for maintaining systemic glucose homeostasis, is essential for providing energy during fasting, famine, and stress situations (Zhao et al. 2023). In physiological homeostasis, the liver synthesizes glucose from non‐carbohydrate precursors, such as lactate, pyruvate, and glucogenic amino acids, via gluconeogenesis, thereby maintaining glucose homeostasis (Xu et al. 2022). Nonetheless, this regulating system is disrupted in cases of lipid metabolism dysregulation, resulting in abnormal overexpression of gluconeogenesis. Lipid metabolism dysregulation is defined by dyslipidemia, ectopic lipid accumulation, and adipose tissue impairment (Dakal et al. 2025). Growing evidence suggests that dysregulation of lipid metabolism stimulates increased gluconeogenesis due to the combined effects of chronic inflammation, compromised insulin signaling, elevated fatty acid oxidation, and heightened metabolic substrates, consequently leading to metabolic disorders such as obesity, diabetes mellitus, and metabolic‐associated fatty liver disease (MAFLD) (He et al. 2025; Yoon et al. 2021).

Chronic inflammation is a fundamental mechanism that promotes the increase of gluconeogenesis caused by lipid metabolism dysregulation. In the context of lipid metabolic dysregulation, ectopic lipid accumulation transpires in vital organs such as the liver, skeletal muscle, and heart. Simultaneously, adipose tissue releases substantial quantities of proinflammatory cytokines, including tumor necrosis factor‐α (TNF‐α), interleukin‐6 (IL‐6), and monocyte chemoattractant protein‐1 (MCP‐1) (Cao et al. 2025). These proinflammatory cytokines may initiate the NF‐κB‐mediated inflammatory signaling cascade, leading to systemic chronic inflammation (Anilkumar and Wright‐Jin 2024) Moreover, they interfere with insulin signaling, cause insulin resistance (IR), and thus enhance the abnormal upregulation of gluconeogenesis, eventually worsening the cycle of metabolic dysregulation (Jung and Choi 2014).

The PI3K/Akt signaling pathway functions as a crucial molecular hub for insulin's physiological effects and is essential for regulating systemic glucose homeostasis. Insulin binds to the insulin receptor (IR), a receptor tyrosine kinase, prompting fast autophosphorylation of the IR β‐subunit, which generates docking sites for insulin receptor substrate‐1 (IRS1). IRS1 is subsequently phosphorylated on tyrosine residues, hence activating class IA phosphoinositide 3‐kinase (PI3K). PI3K facilitates the phosphorylation of phosphatidylinositol‐4,5‐bisphosphate (PIP_2_) at the plasma membrane, resulting in the production of phosphatidylinositol‐3,4,5‐trisphosphate (PIP_3_) (Le et al. 2023). Locally increased PIP_3_ attracts 3‐phosphoinositide‐dependent protein kinase‐1 (PDK‐1) and protein kinase B (AKT) via their respective pleckstrin homology (PH) domains. PDK‐1 subsequently phosphorylates Akt at Thr308 inside its activation loop (Levina et al. 2022). Activated Akt regulates many essential physiological processes, including glycogen synthesis, glucose transport, and apoptosis (Fontana et al. 2024). In the setting of chronic inflammation and insulin resistance, hepatic Akt signaling is compromised, resulting in prolonged nuclear retention of Forkhead box protein O1 (FoxO1). FoxO1 subsequently enhances the transcriptional expression of phosphoenolpyruvate carboxykinase (PEPCK) and glucose‐6‐phosphatase (G6Pase), resulting in dysregulated elevation of hepatic gluconeogenesis (Sajan et al. 2014). This transcriptional dysregulation sustains excessive hepatic glucose production even after meals, establishing the liver as a pivotal metabolic hub in the advancement of fasting hyperglycemia and insulin resistance. The dysregulation of lipid metabolism, which enhances gluconeogenesis, is a fundamental pathogenic factor in the development of insulin resistance. Clarifying the processes behind this process would enhance our comprehension of metabolic dysregulation generated by high‐fat diets and guide methods for its prevention and treatment.

MSS is a historically significant fermented cuisine, made from red chili peppers, tomatoes, and glutinous rice via a dual fermentation process using ancient methods. MSS, rich in lycopene and capsaicin, offers several health advantages: lycopene mitigates diet‐induced obesity and insulin resistance (Zhu et al. 2020), whilst capsaicin ameliorates dysregulated lipid and glucose metabolism caused by high‐fat, high‐sugar diets and fosters gut microbiota reconfiguration (Gong et al. 2022). While MSS contains several functional components that modulate glucose and lipid metabolism, our first findings indicate that its health advantages arise from the copious bioactive chemicals and advantageous molecules produced during fermentation. Collectively, this provide a distinctive food microecology that regulates the biological functioning of MSS (Yuan et al. 2022). Initial experiments demonstrated that MSS enhances lipid metabolic disorders caused by high‐fat diets through the activation of AMP‐activated protein kinase‐alpha (AMPKα), consequently inhibiting the expression of downstream transcription factors sterol regulatory element‐binding protein 1c (SREBP‐1c) and fatty acid synthase (Fasn) (Yang et al. 2021). MSS simultaneously reinstates the number and diversity of gut microbiota in obese mice subjected to high‐fat diets. At the phylum level, there is a rise in Firmicutes and a decrease in Proteobacteria. At the genus level, it inhibits the proliferation of harmful bacteria while promoting the growth of short‐chain fatty acid‐producing bacteria, including Bacteroides and Lactobacillus. MSS diminishes the concentrations of inflammatory mediators TNF‐α and IL‐6 in the intestinal mucosa of obese rats, hence enhancing gut microbiota and lipid metabolism disturbances generated by a high‐fat diet (Zhou et al. 2023). This research seeks to further examine the impact of MSS on enhancing insulin resistance and energy metabolism problems resulting from lipid metabolism abnormalities, along with their underlying processes.

## 2 Results

### 2.1 MSS Alters Body Weight, Glucose‐Lipid Parameters, and Hepatic Morphology in HFD‐Induced Rats

**FIGURE 1:**
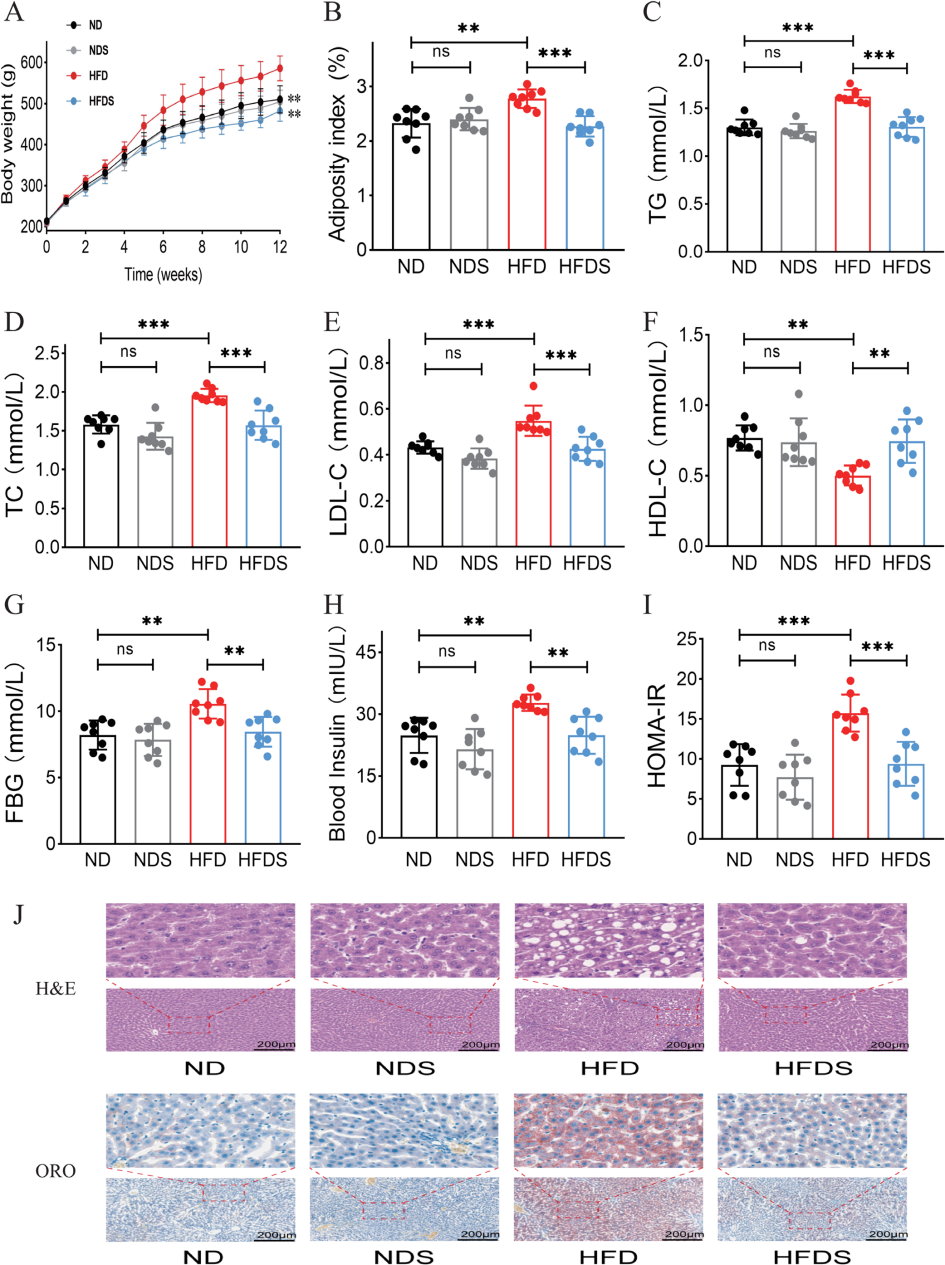
Impact of MSS on overall health in rats induced with high‐fat diet. (A) Variations in body weight among rats over a 12 week period; (B) levels of adiposity index across various groups; (C–F) concentrations of serum triglycerides, total cholesterol, LDL cholesterol, and HDL cholesterol across different groups; (G–I) serum fasting blood glucose, serum insulin levels, and HOMA‐IR in distinct groups; (J) H&E staining and oil red O staining of hepatic tissue in various groups. Values are expressed as the mean ± SEM, *n* = 8. **p < 0.01, ***p < 0.001.

Following a 12—week intervention, the HFD group had increased body weight (Figure 1A) and obesity index (Figure 1B) relative to the ND, NDS, and HFDS groups. Furthermore, serum triglyceride (TG), total cholesterol (TC), and LDL‐cholesterol (LDL‐C) levels were increased, but high‐density lipoprotein cholesterol (HDL‐C) concentration was reduced (*p* < 0.05) (Figure 1C). Simultaneously, serum fasting blood glucose, blood insulin levels, and HOMA‐IR in the high‐fat diet group were elevated compared to the normal diet, normal diet supplemented, and high‐fat diet supplemented groups (*p* < 0.05) (Figure 1D); no significant changes were seen between the normal diet and normal diet supplemented groups for these parameters (*p* > 0.05). Pathological analysis (Figure 1E) demonstrated hepatic steatosis and inflammatory infiltration in hepatocytes of the HFD group, with substantial improvement in the HFDS group subsequent to MSS intervention.

### 2.2 Impact of MSS on the Hepatic Metabolic Profile in HFD‐Induced Rats

**FIGURE 2:**
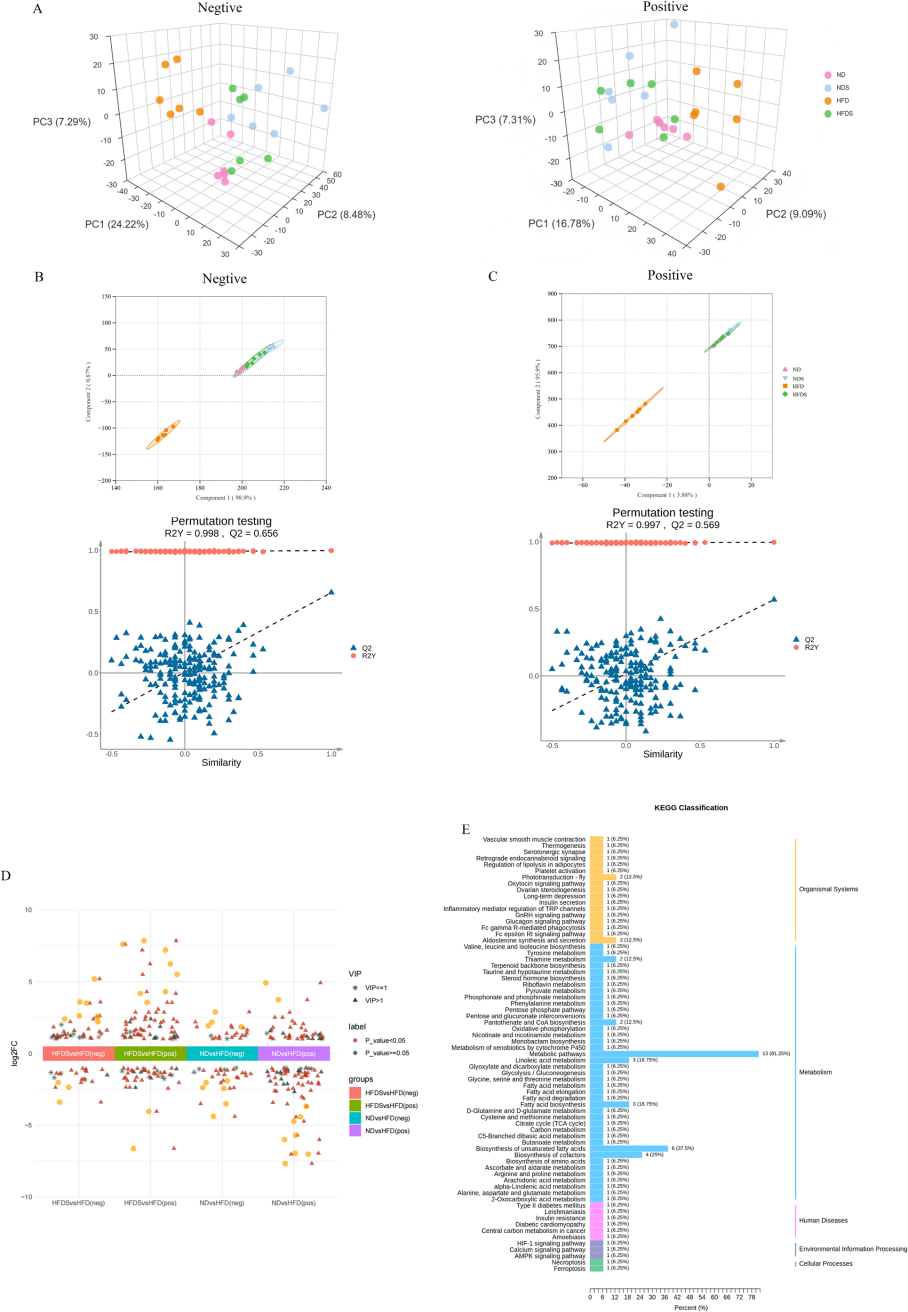
Multivariate statistical examination of rat liver metabolites. (A) PCA analysis conducted in both positive and negative ion modes; (B, C) PLS‐DA analysis with permutation tests (200 iterations) performed in positive and negative ion modes. A goodness of fit (R^2^Y) approaching 1 denotes enhanced model stability and dependability, but a predictive ability (Q^2^) beyond 0.5 shows strong predictive power. (D) Volcano graphs showing differential metabolites across several comparison groups in both positive and negative ion modes. Log_2_FC > 0 signifies upregulation, whereas Log_2_FC < 0 denotes downregulation. (E) KEGG enrichment categorization diagram showing difference metabolites between the HFD and HFDS groups.

We used non‐targeted metabolomics to examine the regulatory function of MSS in hepatic gluconeogenesis generated by a high‐fat diet in rats. A total of 2368 metabolites were identified in positive ion mode, whereas 2591 metabolites were identified in negative ion mode. Principal Component Analysis (PCA) and Partial Least Squares Discriminant Analysis (PLS‐DA) (Figure 2A–C) demonstrated substantial differentiation between the HFD group and the ND, NDS, and HFDS groups, whereas the ND, NDS, and HFDS groups exhibited clustering. Model validation revealed a R^2^Y approaching 1 and a Q^2^ above 0.5, indicating a robust model with independent and reliable data, enabling comprehensive investigation of metabolites at varying abundances.

The PLS‐DA analysis findings yielded the Variable Importance in Projection (VIP), which was determined by the contribution of each variable to intergroup differences. In the PLS‐DA model, a larger VIP value signifies a greater contribution of that variable to the intergroup disparities. Commonly, VIP ≥ 1, *p* < 0.05, and Fold Change ≥ 2 or Fold Change ≤ 0.5 are used as criteria for screening differentiated metabolites. The volcano plot illustrating several differential metabolites (Figure 2D) indicates that, relative to the ND group, the HFD group had 18 upregulated and 21 downregulated metabolites in the negative ion mode, alongside 32 upregulated and 48 downregulated metabolites in the positive ion mode. In comparison to the HFD group, the HFDS group exhibited 24 upregulated and 15 downregulated metabolites in negative ion mode, as well as 47 upregulated and 32 downregulated metabolites in positive ion mode. The enrichment analysis of differential metabolites between the HFD and HFDS groups, conducted using the KEGG database, indicated (Figure 2E) significant enrichment in pathways associated with energy metabolism, including Glycolysis/Gluconeogenesis, Insulin secretion, Pyruvate metabolism, Citrate cycle (TCA cycle), and Glucagon signaling pathway. These findings suggest that MSS intervention may ameliorate energy metabolism disorders in HFD‐induced rats via metabolic pathways such as Glycolysis/Gluconeogenesis.

### 2.3 MSS Improves Chronic Inflammatory Response in HFD‐Induced Rats

**FIGURE 3:**
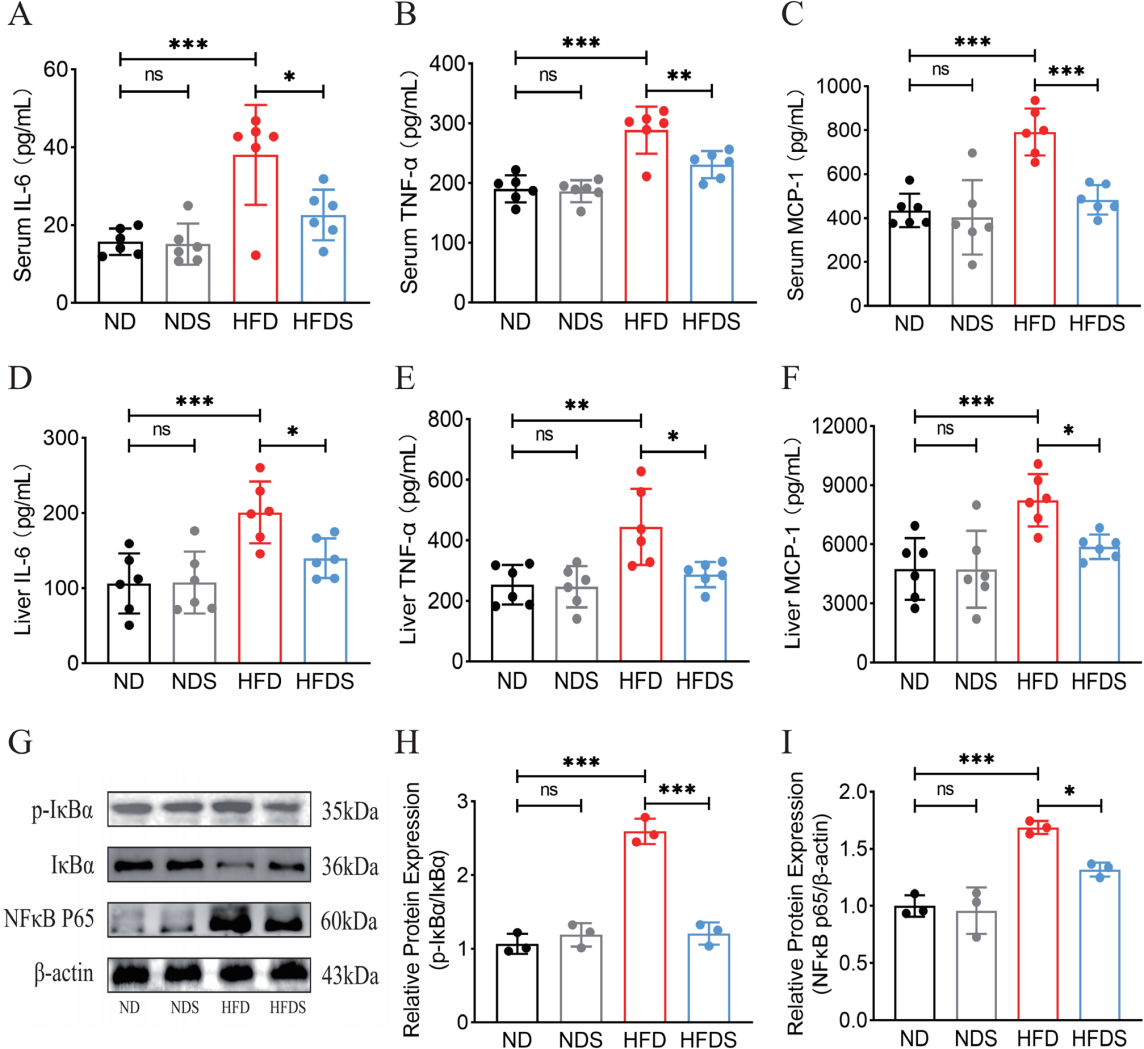
Effect of MSS on inflammation (A–F) The ELISA detection kit detected the expression levels of IL‐6, TNF‐α, and MCP‐1 in rat serum and liver, *n* = 6. (G–I) Western blot detection of protein expression levels of p‐IκBα, IκBα, and NF‐κB in rat liver tissue. Values are expressed as the mean ± SEM, *n* = 3. *p < 0.05, **p < 0.01, ***p < 0.001.

In comparison to the ND group, rats in the HFD group demonstrated increased concentrations of IL‐6, TNF‐α, and MCP‐1 in both serum and hepatic tissues. In contrast, the levels of these inflammatory markers were decreased in the HFDS group (Figure 3A–F). Additionally, p‐IκBα/IκBα and NF‐κB expression were elevated in the livers of the HFD group, whereas p‐IκBα/IκBα was reduced in the HFDS group, resulting in the inhibition of NF‐κB expression (Figure 3G–I). The results demonstrate that MSS reduces chronic inflammatory responses in HFD‐induced rats via suppressing the NF‐κB inflammatory signaling pathway.

### 2.4 MSS Enhances Inhibition of Hepatic Gluconeogenesis by Activating the PI3K/Akt/Foxo1 Signaling Pathway

**FIGURE 4:**
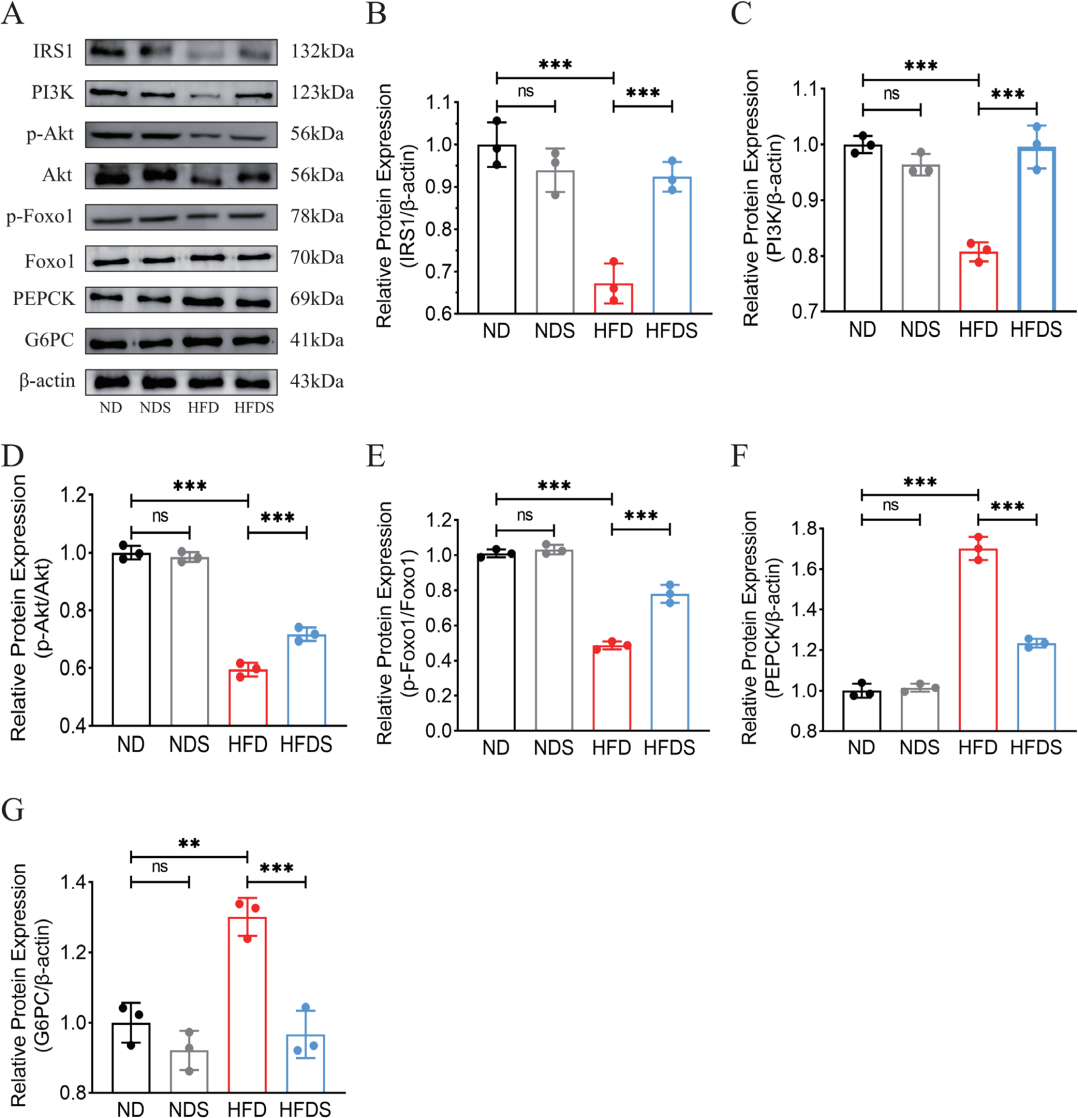
MSS enhances inhibition of hepatic gluconeogenesis by activating the PI3K/Akt/Foxo1 signaling pathway. (A–G) Western blot detection of protein expression levels of IRS1, PI3K, p‐Akt, Akt, p‐Foxo1, Foxo1, PEPCK, and G6PC in rat liver tissue. Values are expressed as the mean ± SEM, *n* = 3. **p < 0.01, ***p < 0.001.

Relative to the ND group, the HFD group demonstrated less IRS1 (insulin receptor substrate‐1) expression, which impeded the downstream PI3K/Akt signaling pathway, resulting in lowered p‐Foxo1/Foxo1 levels and increased expression of PEPCK and G6PC, consequently augmenting gluconeogenesis. In contrast, the HFDS group had heightened IRS1 expression, which stimulated the PI3K/Akt signaling pathway, resulting in higher p‐Foxo1/Foxo1 levels and decreased expression of PEPCK and G6PC. The findings demonstrate that MSS intervention enhances the expression of the insulin receptor IRS1 in the livers of HFD‐fed rats, thereby activating the PI3K/Akt/Foxo1 signaling pathway, increasing the expression of PEPCK and G6PC, and ultimately suppressing gluconeogenesis and ameliorating insulin resistance (Figure 4A–G).

## 3 Discussion

This work comprehensively examined the regulatory function of MSS in glucose metabolic disturbances caused by lipid metabolism dysregulation, along with its interaction with glucose and lipid metabolism. Upon reaching the saturation of adipose tissue storage capacity, surplus lipids are redirected to peripheral tissues, such as the liver, leading to ectopic lipid buildup and a series of metabolic problems, including insulin resistance (Janssen 2024). These metabolic irregularities further increase blood glucose levels, worsening lipid metabolism dysregulation. The liver, as a pivotal organ for glucose and lipid metabolism and a principal target of insulin signaling, demonstrates significantly elevated gluconeogenesis and augmented endogenous glucose synthesis under insulin resistance settings, becoming a prominent contributor to fasting hyperglycemia (Hatting et al. 2018). Simultaneously, excessive hepatic fat buildup induces inflammatory reactions and enhances the release of several inflammatory mediators. These proinflammatory substances impair insulin signaling pathways, worsening hepatic insulin resistance and creating a vicious loop that perpetuates the decline of glucose and lipid metabolic diseases. This cascade ultimately results in metabolic disorders, including diabetes mellitus, obesity, and metabolic‐associated fatty liver disease (MAFLD), which significantly disrupt metabolic homeostasis and general health (Nagai et al. 2010; Ziolkowska et al. 2021).

Multiple studies have emphasized the possible health benefits of fermented foods. MSS is primarily produced from red chili peppers and tomatoes using a conventional two‐stage fermentation method, resulting in a product rich in bioactive compounds including lycopene, capsaicin, and short‐chain fatty acids. Lycopene, recognized for its antioxidant properties, has been associated with the treatment of cancer, infertility, metabolic syndrome, and hepatic damage. Capsaicin, the primary bioactive component of chili peppers, stimulates lipid metabolism via the activation of peroxisome proliferator‐activated receptor gamma (PPARγ) and has anti‐inflammatory and antioxidant properties. Lactic acid, acetic acid, and citric acid have shown anti‐inflammatory properties, improved immunological tolerance, safeguarded neurons, and mitigated ketosis in rats (Maruta et al. 2022; S. Sun et al. 2017). Research results demonstrate that MSS intervention decreased body weight in HFD‐induced rats, enhanced blood glucose, blood lipids, insulin levels, and HOMA‐IR, while also mitigating hepatic fat accumulation. This indicates that MSS may alleviate HFD‐induced disturbances in glucose and lipid metabolism and diminish insulin resistance. To clarify the fundamental processes, we discovered liver metabolites in rats across several groups using UPLC‐QTOF‐MS. PCA and PLS‐DA analysis demonstrated distinctions between the ND group and the HFD group, as well as between the HFDS group and the HFD group, with the latter exhibiting a tendency more akin to the ND group. This suggests that a high‐fat diet causes metabolic problems in rats, which MSS may alleviate. No changes were detected between the ND and NDS groups, suggesting that MSS did not negatively impact rats in normal physiological circumstances. The subsequent examination of differential metabolites and KEGG enrichment indicated that energy metabolic pathways, including Glycolysis/Gluconeogenesis and Insulin secretion, were modulated by MSS between the HFD and HFDS groups. We propose that the beneficial effects of MSS on glucose and lipid metabolism diseases, as well as insulin resistance generated by a high‐fat diet, are linked to these energy metabolism pathways. Subsequent analysis indicated that the health benefits of MSS on glucose and lipid metabolism may be influenced by its impact on chronic inflammation and the regulation of the PI3K/Akt/Foxo1 pathway.

The NF‐κB signaling pathway as a principal regulator of inflammatory reactions. The IκBα protein, in its inactive form, associates with the NF‐κB dimer, sequestering it inside the cytoplasm and inhibiting the nuclear translocation of the NF‐κB p65 subunit (Ghosh and Karin 2002). In contrast, following conventional activation of the NF‐κB pathway, IκBα is swiftly degraded, allowing the NF‐κB dimer to translocate to the nucleus, where it binds to DNA and activates immune response‐related genes (Vallabhapurapu and Karin 2009). This study's findings indicate that a high‐fat diet triggers low‐grade chronic inflammation in rats, marked by increased levels of proinflammatory cytokines (e.g., IL‐6, TNF‐α, and MCP‐1), degradation of hepatic IκBα, and subsequent activation of the NF‐κB pathway. MSS intervention markedly reduced IκBα degradation, obstructed nuclear translocation of the NF‐κB protein, and therefore downregulated the expression of NF‐κB‐mediated proinflammatory signaling pathways, resulting in decreased circulatory proinflammatory cytokine levels. The findings demonstrate that MSS efficiently alleviates chronic inflammatory responses generated by a high‐fat diet in rats by inhibiting the activation of the NF‐κB signaling pathway.

The PI3K/Akt pathway serves as the primary signaling axis facilitating insulin action. Insulin interaction to the insulin receptor induces phosphorylation of serine residues on IRS‐1, subsequently activating downstream Akt. Nonetheless, this pathway is compromised in chronic inflammatory circumstances, resulting in the IRS‐1‐mediated downregulation of PI3K and the consequent suppression of Akt activation (Gao et al. 2002). The inhibition of the PI3K/Akt pathway is a defining characteristic of systemic insulin resistance, as it disrupts insulin's normal signaling through IRS‐1 in hepatocytes, hindering transmembrane glucose transport and cellular glucose utilization, ultimately resulting in increased circulating glucose levels (Y. Liu et al. 2019). This pathway is a crucial regulator of hepatic gluconeogenesis, influencing the activity of transcription factors such peroxisome proliferator‐activated receptor gamma coactivator 1α (PGC‐1α) and FoxO1 (Gu et al. 2019). These transcription factors facilitate glucose homeostasis by modulating essential gluconeogenic enzymes, such as PEPCK and G6Pase (Barthel and Schmoll 2003).

Research has shown that the PI3K/Akt pathway is a major regulator of the downstream Foxo1 protein, an essential transcription factor in glucose metabolism. The suppression of Foxo1 expression is directly associated with reduced hepatic gluconeogenesis and improved glucose metabolism in animal models of type 2 diabetes (Q. Liu et al. 2017). In the liver, Foxo1 is activated during fasting, resulting in the upregulation of PEPCK and G6PC expression; conversely, postprandially, Foxo1 is inactivated, causing a reduction in PEPCK and G6PC levels—this represents a principal mechanism through which insulin swiftly and efficiently inhibits hepatic gluconeogenesis following food intake (Lu et al. 2012). PEPCK and G6PC serve as rate‐limiting enzymes in gluconeogenesis. PEPCK facilitates the transformation of oxaloacetate into phosphoenolpyruvate, which then undergoes a sequence of processes to produce glucose‐6‐phosphate; G6PC dephosphorylates glucose‐6‐phosphate to glucose, permitting its unrestricted release from cells into the bloodstream (Shah and Wondisford 2020). This research reveals that during systemic lipid metabolic dysregulation, adipose tissue secretes excessive proinflammatory cytokines, such as IL‐6, TNF‐α, and MCP‐1. These cytokines stimulate the NF‐κB signaling pathway, impair insulin signaling, and eventually promote insulin resistance. The fundamental mechanism encompasses the following elements: activation of the NF‐κB signaling pathway in rat livers due to a high‐fat diet results in decreased IRS‐1 expression, which inhibits the PI3K/Akt/Foxo1 signaling pathway and increases downstream expression of PEPCK/G6PC, ultimately enhancing hepatic gluconeogenesis and elevating blood glucose levels; Conversely, subsequent to MSS intervention, the NF‐κB signaling pathway is inhibited, and IRS‐1 expression is enhanced, which activates the PI3K/Akt/Foxo1 signaling pathway and diminishes the expression of PEPCK/G6PC. These actions jointly diminish hepatic gluconeogenesis and alleviate insulin resistance.

Our results indicate that Foxo1, as a downstream effector of the PI3K/Akt signaling pathway, modulates PEPCK/G6PC expression. In the setting of high‐fat diet‐induced dysregulation of lipid metabolism, Foxo1 significantly exacerbates disturbances in glucose metabolism. In HFD‐induced rats, MSS may reduce aberrant hepatic gluconeogenesis, preserve glucose homeostasis, and improve insulin resistance via lowering chronic inflammation and modulating the PI3K/Akt/Foxo1 signaling pathway. The potential health‐enhancing attributes of MSS, a traditional fermented food, may provide hope for the management of metabolic diseases. Nonetheless, its particular advantageous benefits on the general population have yet to be clarified. In future research, we will do population‐based investigations to further corroborate and expand these results.

## 4 Materials and Methods

### 4.1 Preparation of Miao Sour Soup

MSS (Food License No. SC12452263500060) was purchased from Mingyang Foods Co. Ltd. (Guizhou, China). Prepare a dilution of the MSS with distilled water to achieve a concentration of 0.8 g/mL. Heat the diluted MSS to its boiling point, then chill and filter for further use. According to the dietary intake survey, the common dose of MSS is about 10 g/500 mL for adults, and the equivalent dose for rats and humans was converted to determine the MSS intervention dose of 8 g/kg BW per day for this study (Conversion multiplier for 200 g rat to human is 0.16 times) (Yuan et al. 2022).

### 4.2 Animal Model

Sprague Dawley (SD) rats were provided by Guizhou Medical University. This study was approved by the Ethical Review Committee for Laboratory Animals of Guizhou Medical University (Grant No. 2100210). These rats were housed at a temperature of 20°C–25°C, with a humidity of 50%–70% and a 12‐h light/dark cycle. After a week of acclimatization feeding, all rats were divided into four groups as follows (*n* = 8): ND (normal feed + distilled water), NDS (normal feed + MSS), HFD (high fat feed + distilled water), HFDS group (high fat feed + MSS). The feed was provided by according to the preexisting experiments. Gavage intervention (intervention dose: 8 g/kg BW) was administered to all groups of rats at exactly 3:00 p.m. each day for 12 weeks. The body weights of the rats in each group were recorded weekly. After the completion of Week 12, the rats had a 12‐h fasting period. The subjects were then terminated by intraperitoneal injection of sodium pentobarbital (0.5 mL/100 g body weight), and blood samples were obtained. Subsequently, intra‐abdominal adipose and liver tissues were dissected to remove them and weighed for subsequent testing. Adiposity index were calculated according to Yang's method (Yang et al. 2021).

### 4.3 Biochemical Analysis

Blood samples were centrifuged (TDL‐5000bR, China) at 4°C, 3000 r/min for 15 min to obtain serum, which was stored at −20°C. Serum triglycerides, total cholesterol, low‐density lipoprotein cholesterol (LDL‐C), high‐density lipoprotein cholesterol (HDL‐C), and fasting blood glucose levels were measured using a Beckman Coulter biochemical analyzer (Lx‐20, Brea). Serum insulin was detected using a rat insulin ELISA kit (Solarbio, China), and the insulin resistance index, HOMA‐IR, was calculated according to the method of J. Sun et al. (2024). Serum and liver IL‐6, TNF‐α, and MCP‐1 levels were measured using ELISA kits (Solarbio, China).

### 4.4 Pathological‐Histological Examination

Liver tissue was preserved in 10% paraformaldehyde (McLean Biological, China). H&E staining: Tissue was dehydrated with alcohol, paraffin‐embedded, sectioned (4 μm thick), stained with hematoxylin–eosin (McLean Biological, China) for 5 min, then dehydrated with alcohol. Pathological alterations in hepatic tissue were examined using a light microscope (Olympus, Japan) at 100× and 400× magnification. Oil Red O staining: Tissue was dehydrated, cryosectioned, washed with 60% isopropanol, stained with Oil Red O (McLean Biological, China) solution for 10 min, differentiated, counterstained with Mayer's eosin for 2 min, and blued. Lipid accumulation in hepatic tissue was seen using a light microscope (Olympus, Japan) at 200× and 400× magnification.

### 4.5 Non‐Targeted Metabolomics Analysis

#### 4.5.1 Preliminary Treatment of Hepatic Tissue Specimens

Take out the sample from the −80°C refrigerator and thaw it on ice. Multi‐point sample and weigh 20 mg of sample, homogenize (30 HZ) for 20 s with a steel ball and centrifuge (3000 rpm, 4°C) for 30 s. Then add 400 μL of 70% methanol water internal standard extractant, shake (1500 rpm) for 5 min and place on ice for 15 min. Centrifuge (12,000 rpm, 4°C) for 10 min, transfer 300 μL of the supernatant and let it stand still at −20°C for 30 min. Finally, centrifuge (12,000 rpm, 4°C) for 3 min and take the supernatant for analysis.

#### 4.5.2 HPLC Conditions (T3)

All samples were obtained by the LC–MS technique in accordance with machine directives. The analytical parameters were as follows: UPLC column, Waters ACQUITY UPLC HSS T3 C18 (1.8 μm, 2.1 mm × 100 mm); column temperature, 40°C; flow rate, 0.4 mL/min; injection volume, 2 μL; solvent system, water (0.1% formic acid) and acetonitrile (0.1% formic acid); gradient program, 95:5 V/V at 0 min, 10:90 V/V at 11.0 min, 10:90 V/V at 12.0 min, 95:5 V/V at 12.1 min, 95:5 V/V at 14.0 min.

#### 4.5.3 Multivariate Statistical Analysis and Differential Metabolite Profiling

Principal Component Analysis (PCA) and Partial Least Squares Discriminant Analysis (PLS‐DA) were conducted using the ropls package (www.r‐project.org) in R. Before analysis, the data was subjected to unit variance normalization. Metabolites with substantial intergroup regulation were selected using VIP ≥ 1, absolute Log2FC (fold change) ≥ 1, and *p*‐value < 0.05. VIP values were derived from PLS‐DA outcomes, including score plots and permutation plots produced by the MetaboAnalystR R package. The data was subjected to PLS‐DA analysis after log2 transformation and mean centering. Permutation testing (200 permutations) was used to mitigate overfitting.

#### 4.5.4 KEGG Enrichment Analysis

Identified metabolites were annotated using KEGG Compound database (http://www.kegg.jp/kegg/compound/), annotated metabolites were then mapped to KEGG Pathway database (http://www.kegg.jp/kegg/pathway.html). Significantly enriched pathways are identified with a hypergeometric test's *p*‐value for a given list of metabolites.

### 4.6 Western‐Blot Assay

The specific primary antibodies used in this study were p‐IκBα, IκBα, NF‐κB, IRS1, PI3K, p‐Akt, Akt, Foxo1, PEPCK, β‐actin (cat: K009559P, K000498P, K200045M, K001623P, K001787P, K006214P, K002111P, K009562P, K007662P, K200058M, Solarbio, China), G6PC (cat: SL13386R, Sunlong Biotech, China) and p‐Foxo1 (cat: P00073‐4, Boster Biological Technology, China). Take 50–100 mg of liver tissue into the homogenizer, add a mixture of lysate and PMSF for homogenization, ultrasonic crushing and then aspirate the supernatant, dilute it and then carry out protein quantification and protein denaturation in 100°C. Following this, polyacrylamide gel electrophoresis was carried out, transfer the membrane (300 mA, 1 h, 0°C), and then the membrane was closed in a 5% skimmed milk powder TBS‐T solution for 2 h, eluted with TBS‐T solution, incubated with primary antibody overnight, incubated with secondary antibody after elution with TBS‐T solution, exposed after TBS‐T elution.

### 4.7 Statistical Analysis

SPSS22.0 software was used to analyze the differences in body weight, lipids, blood glucose, and inflammatory factors between the groups. One‐way ANOVA was used for homogeneity of variance when the data showed the normal distribution. When the one‐way ANOVA was significant, the least significant difference was used to further analyze the differences between each of any two groups. *p < 0.05, **p < 0.01, ***p < 0.001.

## 5 Conclusions

This research demonstrates that Miao Sour Soup (MSS) mitigates glucose and lipid metabolic abnormalities generated by a high‐fat diet (HFD) in rats. Foxo1, a crucial regulatory protein in lipid metabolism, subsequently influences glucose metabolism. By controlling hepatic gluconeogenesis and downregulating PI3K/Akt/Foxo1‐mediated PEPCK/G6PC via a suppression of the NF‐κB inflammatory signaling cascade, MSS may alleviate insulin resistance.

## Author Contributions

**Xue Ao:** conceptualization, methodology, software, validation, formal analysis, data curation, writing – original draft, visualization, supervision. **Xiaoying Liu:** conceptualization, methodology, software, validation, formal analysis, data curation, writing – original draft, writing – review and editing, visualization. **Xuan Guo:** validation. **Hangning Qian:** validation. **Huihui Li:** methodology. **Qin Yuan:** validation. **Huiqun Wang:** conceptualization, resources, writing – review and editing, supervision, project administration, funding acquisition.

## Funding

This work was supported by the First‐Class Discipline Construction Project in Guizhou Province – Public Health and Preventive Medicine (No. 2017[85]).

## Conflicts of Interest

The authors declare no conflicts of interest.

## Supporting information


**Data S1:** fsn371900‐sup‐0001‐Supinfo.zip.

## Data Availability

The original contributions presented in this study are included in the article/Supporting Information; further inquiries can be directed to the corresponding author.
