# Supplementary figures and images for: Miao Sour Soup Modulates Hepatic Gluconeogenesis Through PI3K/Akt/Foxo1 Signaling in High‐Fat Diet–Induced Rats
Source: Food Sci Nutr. 2026 May 20;14(5):e71900. doi: 10.1002/fsn3.71900 (PMC13238524; doi:10.1002/fsn3.71900)

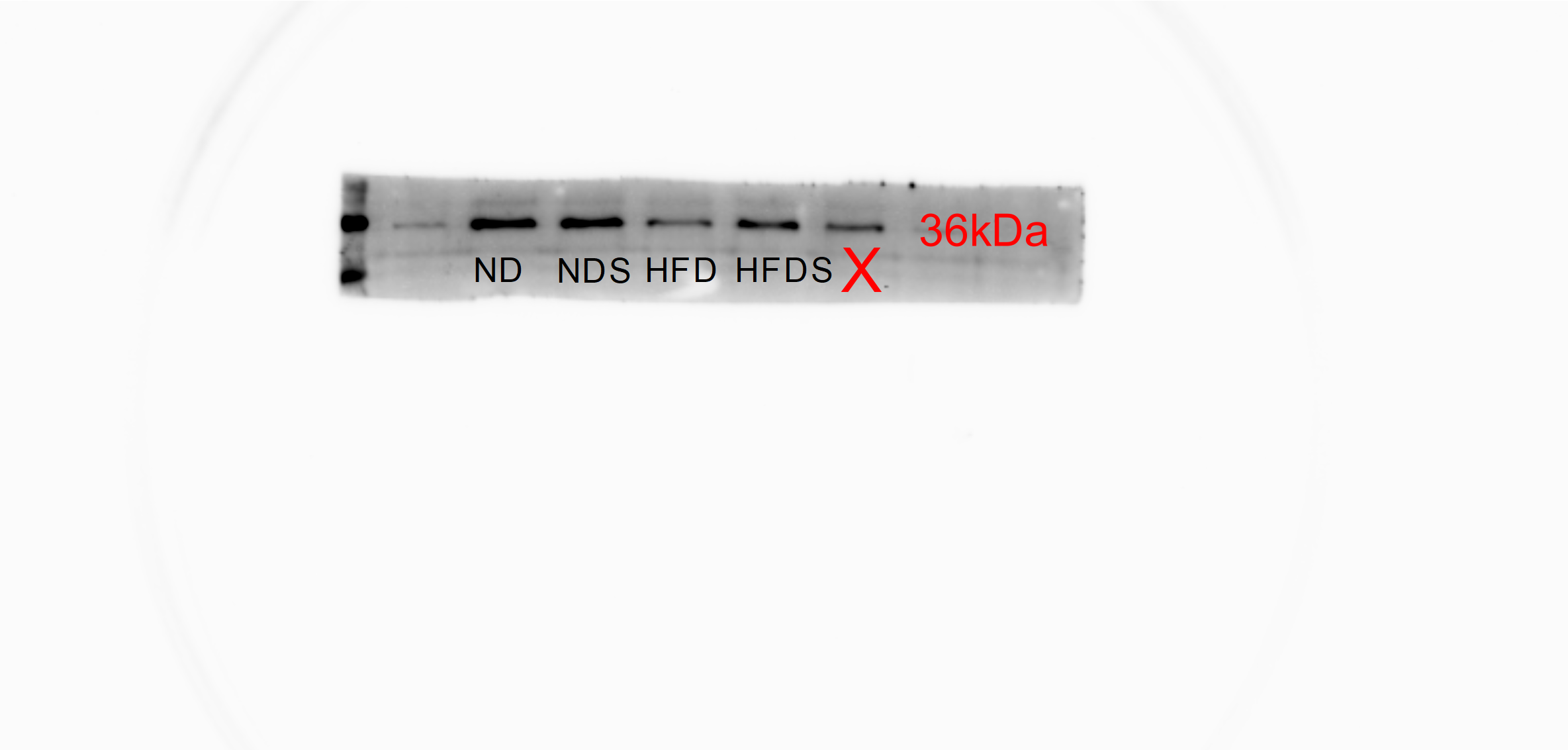

Supplement: Supplementary file 1 — Data S1: fsn371900‐sup‐0001‐Supinfo.zip. [file FSN3-14-e71900-s001.zip › ikba.tif]

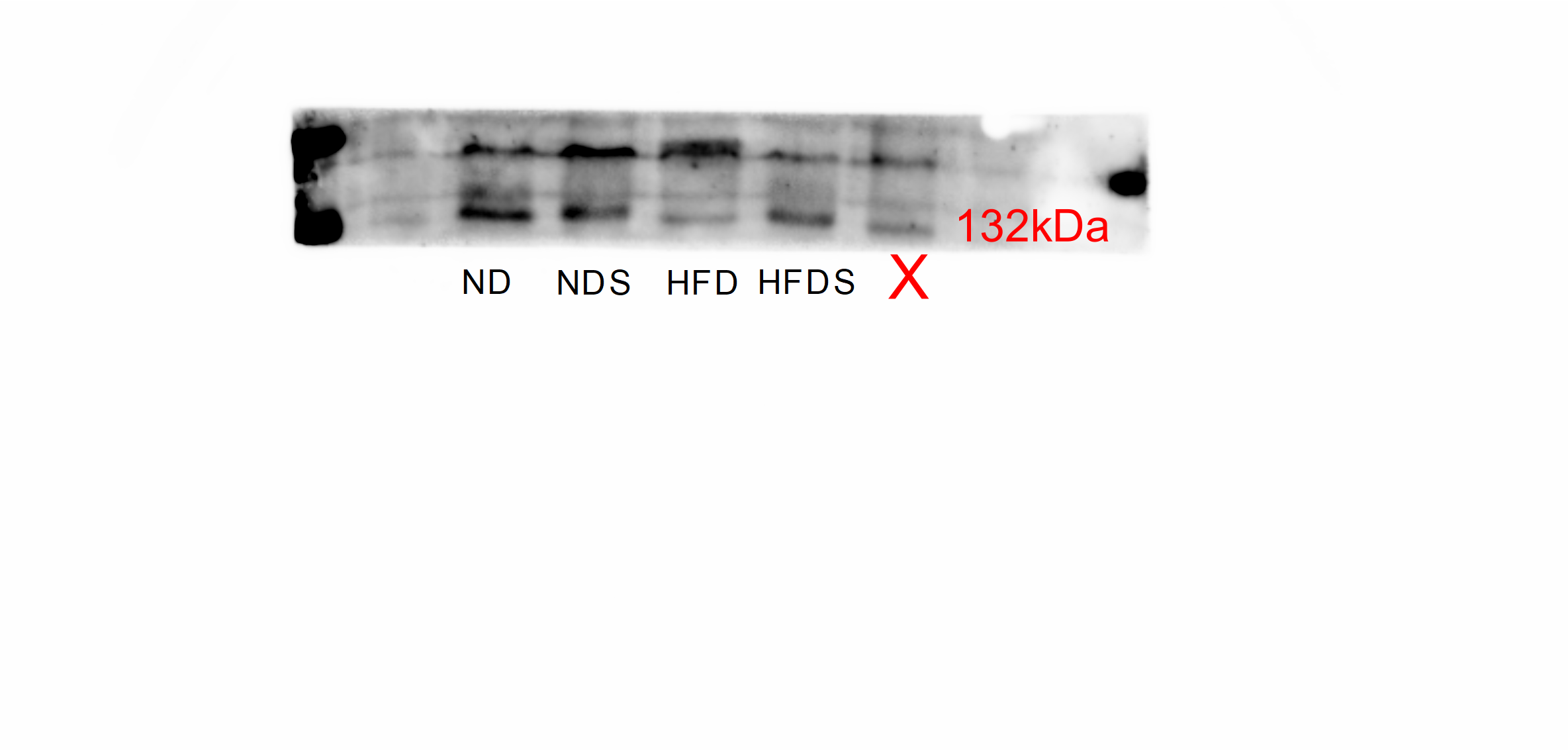

Supplement: Supplementary file 1 — Data S1: fsn371900‐sup‐0001‐Supinfo.zip. [file FSN3-14-e71900-s001.zip › irs1.tif]

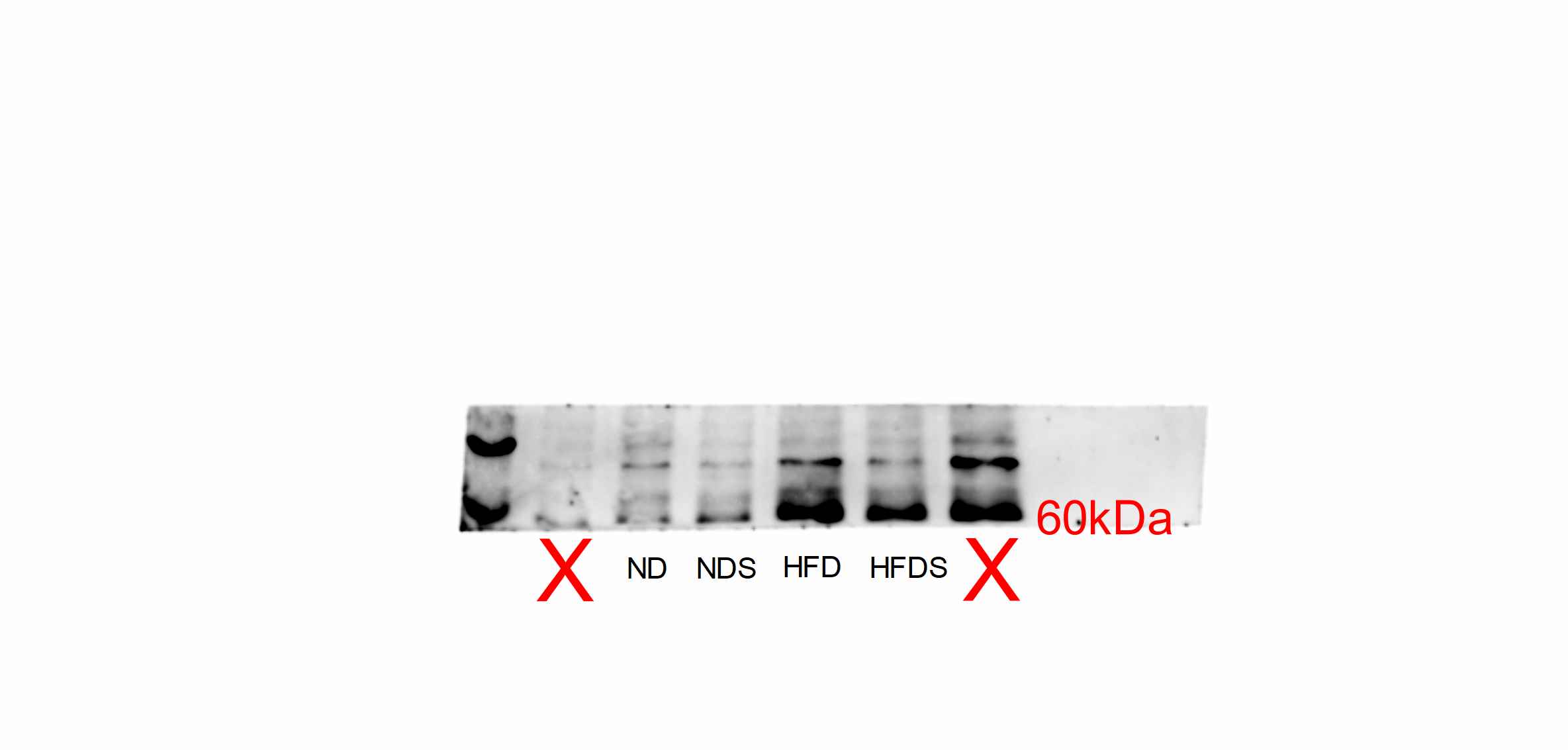

Supplement: Supplementary file 1 — Data S1: fsn371900‐sup‐0001‐Supinfo.zip. [file FSN3-14-e71900-s001.zip › nfkb.tif]

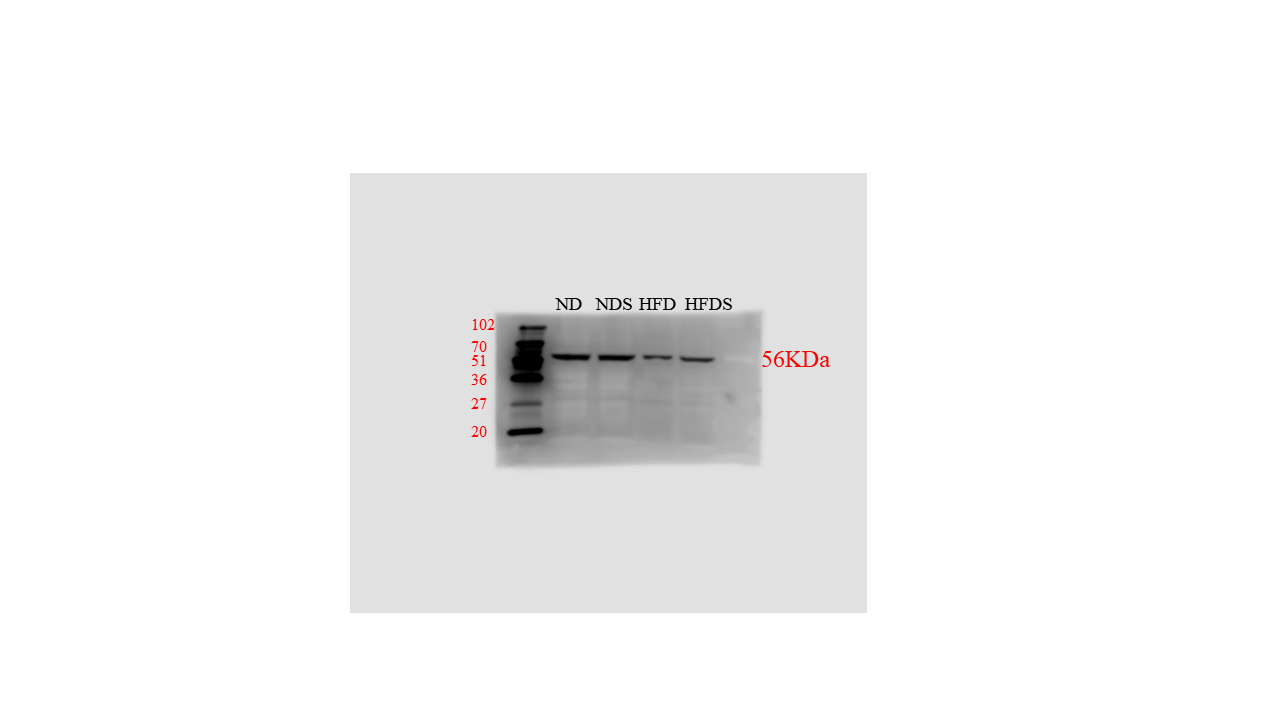

Supplement: Supplementary file 1 — Data S1: fsn371900‐sup‐0001‐Supinfo.zip. [file FSN3-14-e71900-s001.zip › p-Akt.png]

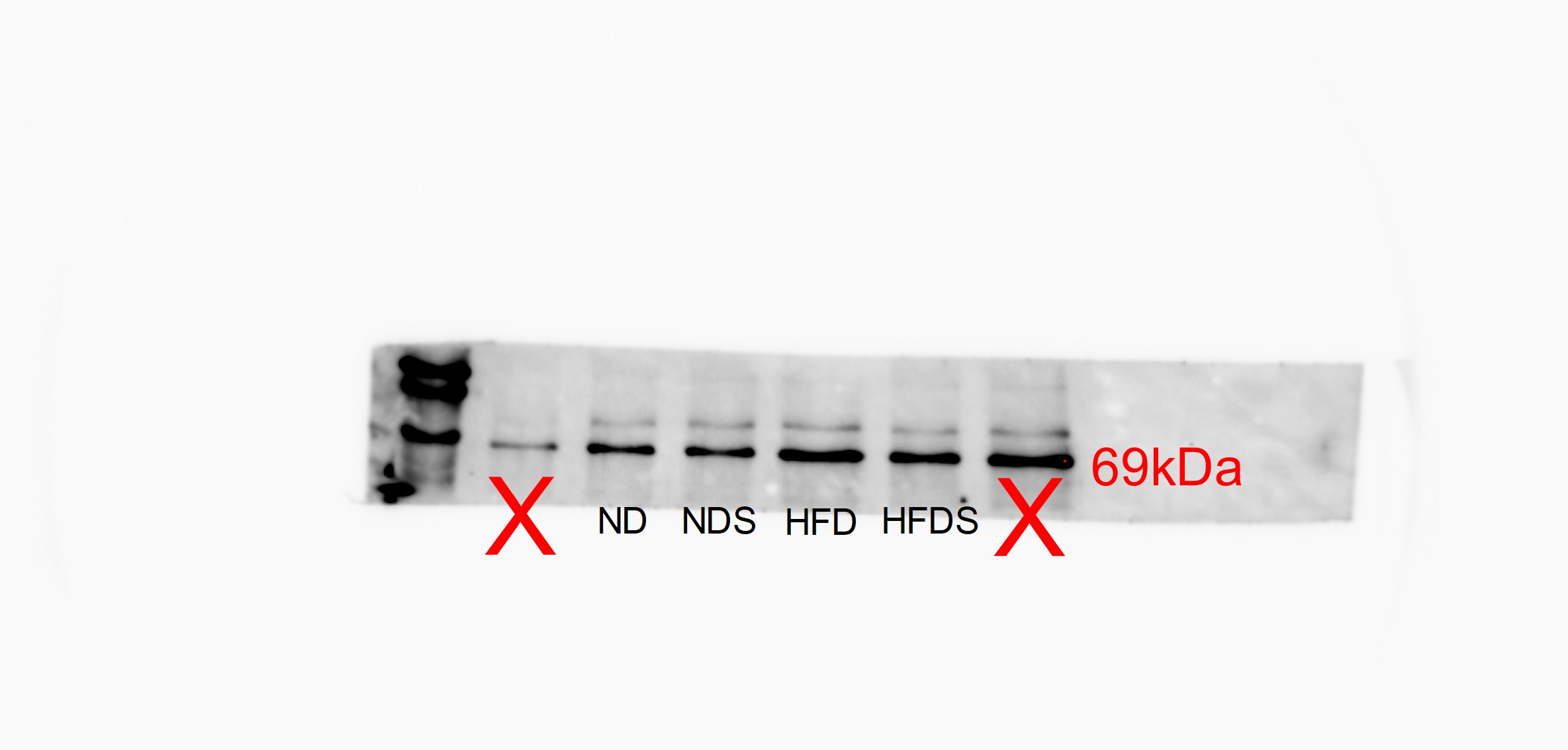

Supplement: Supplementary file 1 — Data S1: fsn371900‐sup‐0001‐Supinfo.zip. [file FSN3-14-e71900-s001.zip › pepck.tif]

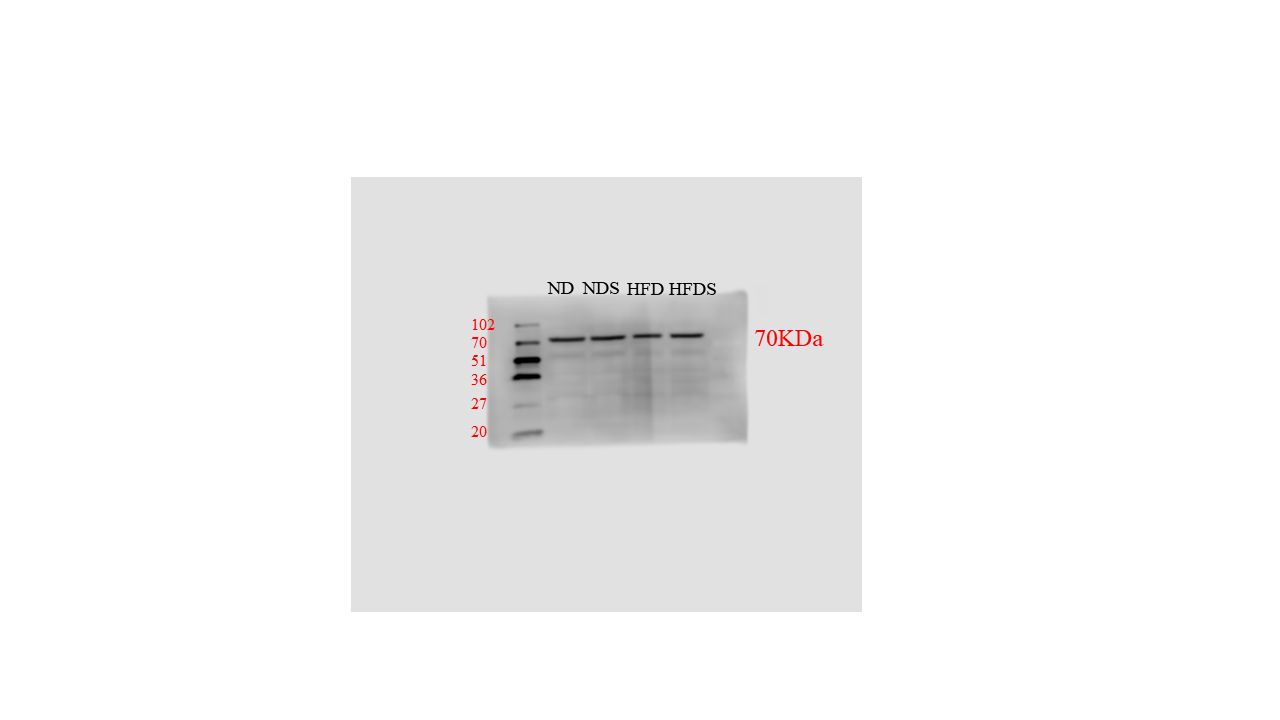

Supplement: Supplementary file 1 — Data S1: fsn371900‐sup‐0001‐Supinfo.zip. [file FSN3-14-e71900-s001.zip › p-Foxo1.png]

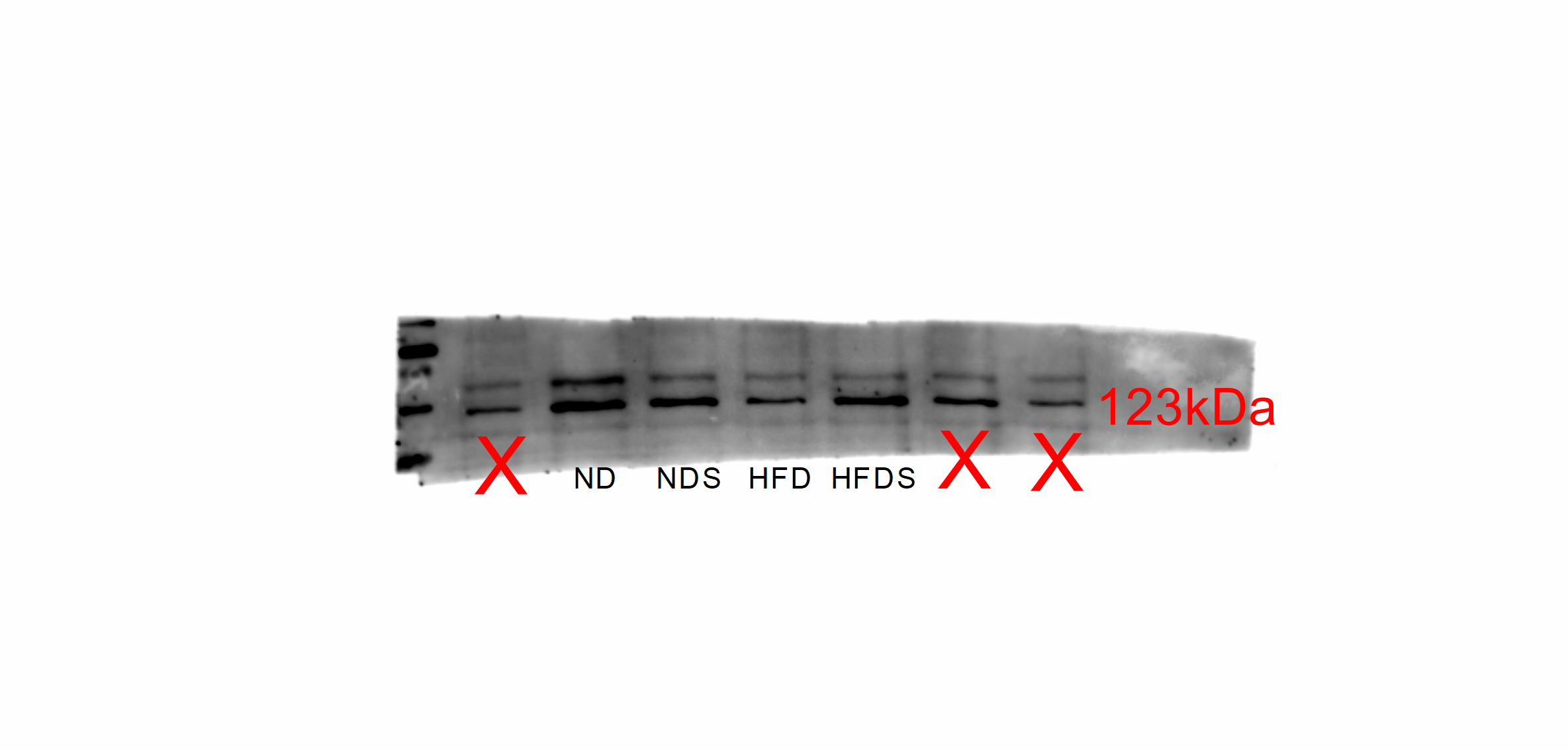

Supplement: Supplementary file 1 — Data S1: fsn371900‐sup‐0001‐Supinfo.zip. [file FSN3-14-e71900-s001.zip › pi3k.tif]

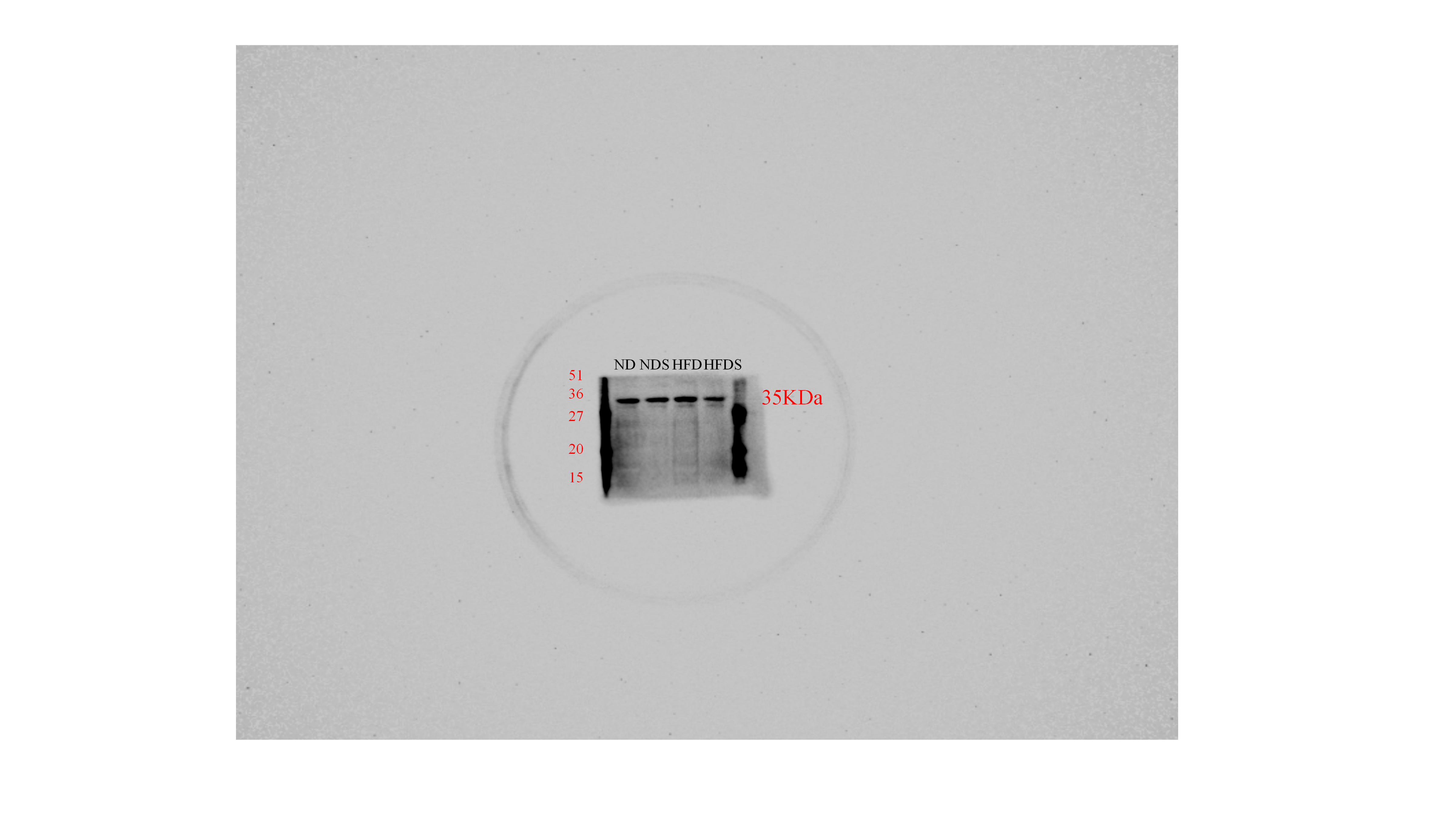

Supplement: Supplementary file 1 — Data S1: fsn371900‐sup‐0001‐Supinfo.zip. [file FSN3-14-e71900-s001.zip › p-ikkb.png]

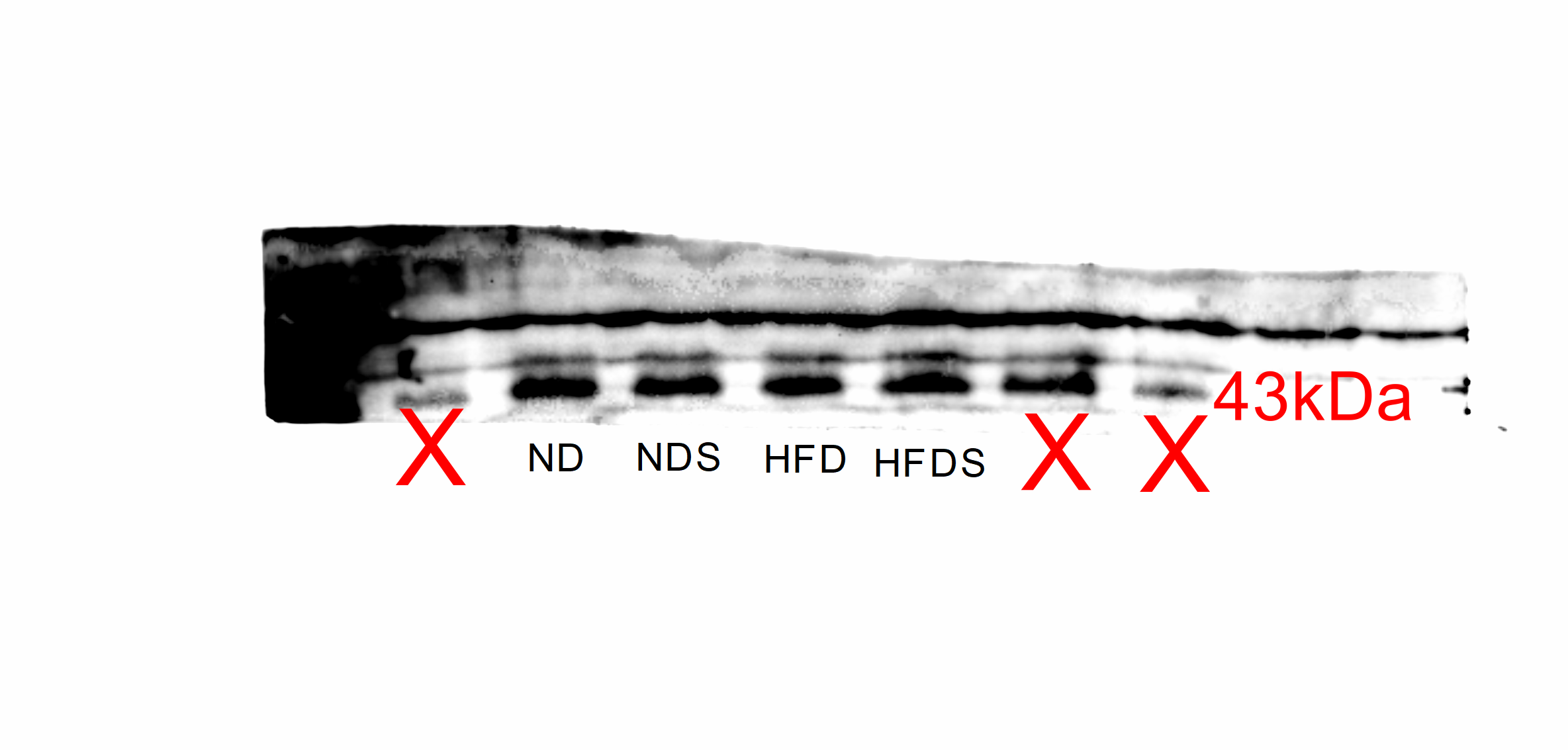

Supplement: Supplementary file 1 — Data S1: fsn371900‐sup‐0001‐Supinfo.zip. [file FSN3-14-e71900-s001.zip › β actin 1.tif]

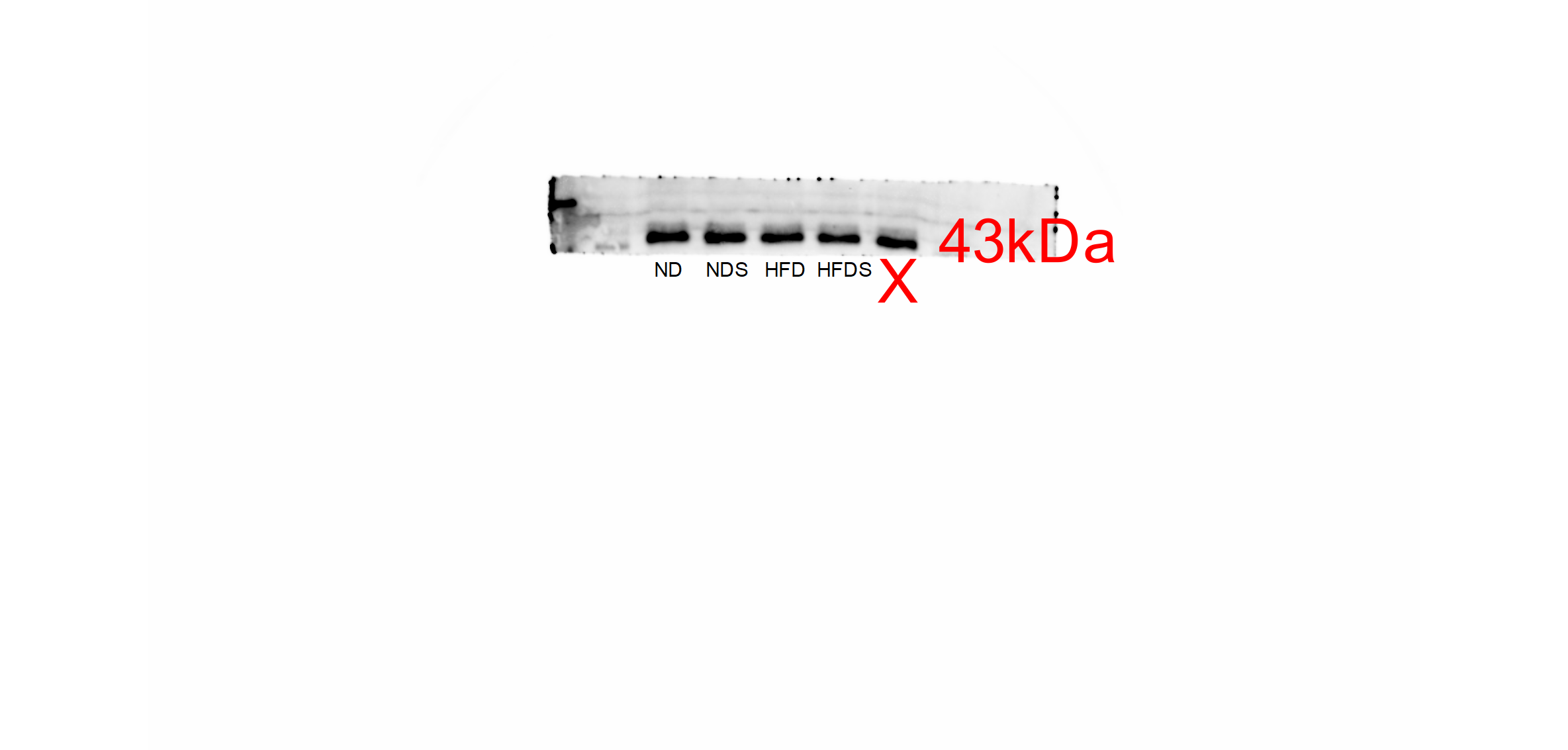

Supplement: Supplementary file 1 — Data S1: fsn371900‐sup‐0001‐Supinfo.zip. [file FSN3-14-e71900-s001.zip › β actin2.tif]

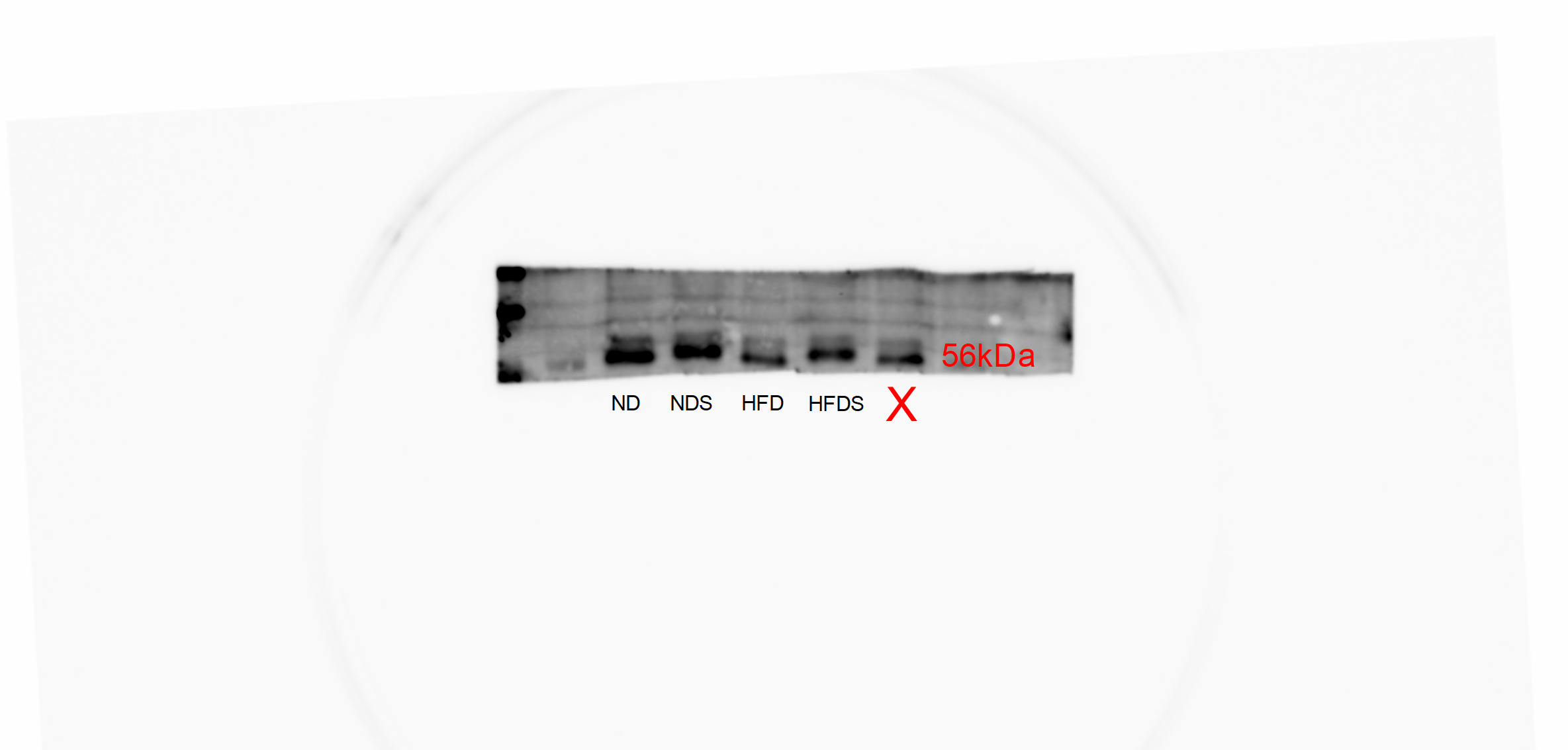

Supplement: Supplementary file 1 — Data S1: fsn371900‐sup‐0001‐Supinfo.zip. [file FSN3-14-e71900-s001.zip › Akt.tif]

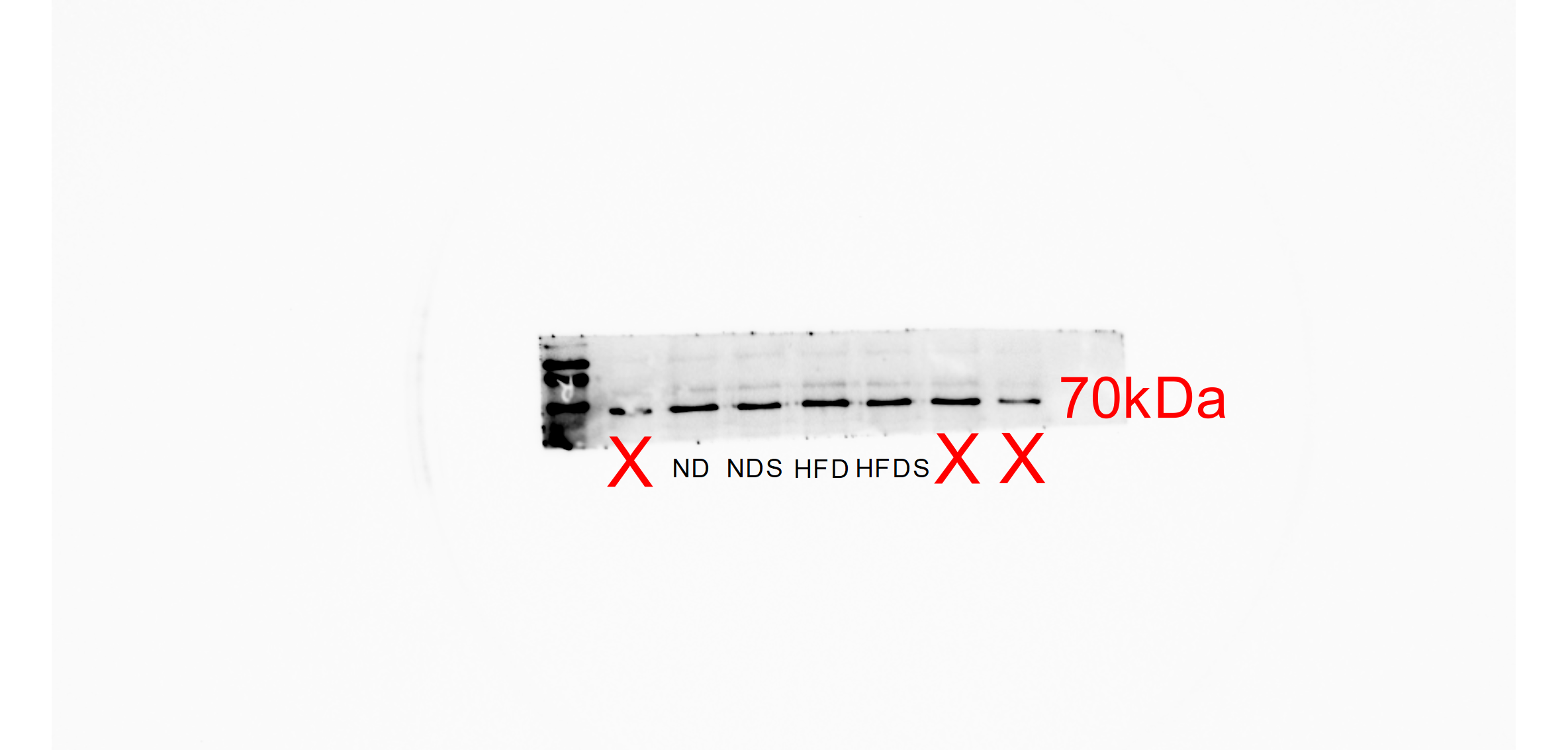

Supplement: Supplementary file 1 — Data S1: fsn371900‐sup‐0001‐Supinfo.zip. [file FSN3-14-e71900-s001.zip › foxo1.tif]

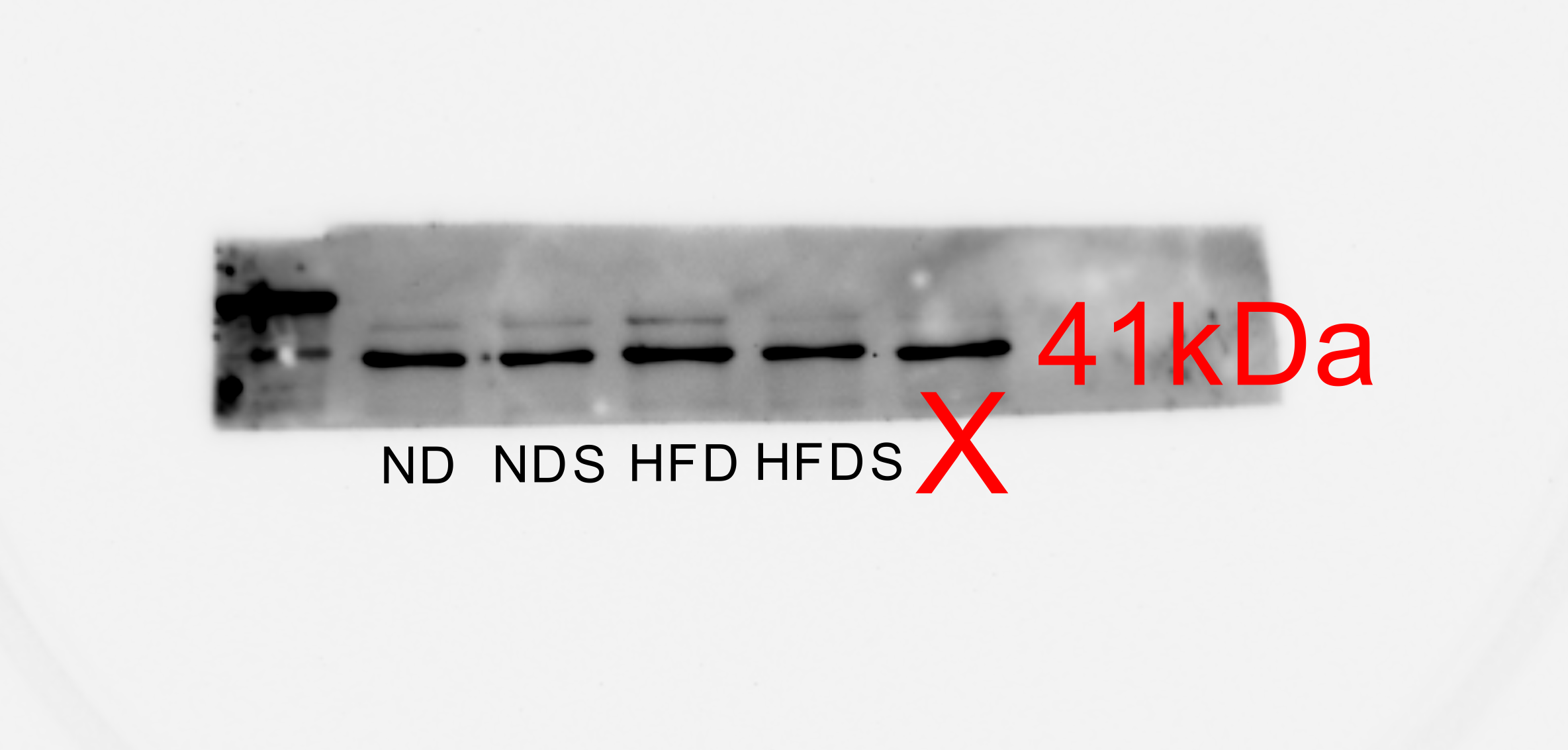

Supplement: Supplementary file 1 — Data S1: fsn371900‐sup‐0001‐Supinfo.zip. [file FSN3-14-e71900-s001.zip › g6pc.tif]
